# Low infection rates after 34,361 intramedullary nail operations in 55 low- and middle-income countries: Validation of the Surgical Implant Generation Network (SIGN) Online Surgical Database

**DOI:** 10.3109/17453674.2011.636680

**Published:** 2011-11-25

**Authors:** Sven Young, Stein Atle Lie, Geir Hallan, Lewis G Zirkle, Lars B Engesæter, Leif I Havelin

**Affiliations:** ^1^Department of Orthopaedic Surgery, Haukeland University Hospital, Bergen; ^2^Department of Surgical Sciences, University of Bergen; ^3^The Norwegian Arthroplasty Register, Bergen, Norway; ^4^The Surgical Implant Generation Network (SIGN), Richland, WA, USA

## Abstract

**Background:**

The Surgical Implant Generation Network (SIGN) supplies intramedullary (IM) nails for the treatment of long bone fractures free of charge to hospitals in low- and middle-income countries (LMICs). Most operations are reported to the SIGN Online Surgical Database (SOSD). Follow-up has been reported to be low, however. We wanted to examine the pattern of follow-up and to assess whether infection rates could be trusted.

**Patients and methods:**

The SOSD contained 36,454 IM nail surgeries in 55 LMICs. We excluded humerus and hip fractures, and fractures without a registered surgical approach. This left 34,361 IM nails for analysis. A generalized additive regression model (gam) was used to explore the association between follow-up rates and infection rates.

**Results:**

The overall follow-up rate in the SOSD was 18.1% (95% CI: 17.7–18.5) and national follow-up rates ranged from 0% to 74.2%. The overall infection rate was 0.7% (CI: 0.6–0.8) for femoral fractures and 1.2% (CI: 1.0–1.4) for tibial fractures. If only nails with a registered follow-up visit were included (n = 6,224), infection rates were 3.5% (CI: 3.0–4.1) for femoral fractures and 7.3% (CI: 6.2–8.4) for tibial fractures. We found an increase in infection rates with increasing follow-up rates up to a level of 5%. Follow-up above 5% did not result in increased infection rates.

**Interpretation:**

Reported infection rates after IM nailing in the SOSD appear to be reliable and could be used for further research. The low infection rates suggest that IM nailing is a safe procedure also in low- and middle-income countries.

Approximately 2.6 million people between the ages of 10 and 24 died globally in 2004. 97% of these lived in low- and middle-income countries (LMICs). 259,000 people in the same age group died in traffic accidents alone. 22% of all deaths in young people are a result of injury, twice as many as those from HIV/AIDS and tuberculosis combined (Patton et al. 2009). For every death resulting from injury, one can expect 3–50 times as many people living with disability as a result of the same injury (Kobusingye et al. 2001, Peden 2004, Gosselin et al. 2009b). Many of these deaths and disabilities could be prevented with better surgical trauma care. However, the funding of this has been neglected by policy makers and international donors, who in previous decades have focused almost entirely on the prevention of communicable disease and primary care (Debas et al. 2006, Mock and Cherian 2008, Ozgediz and Riviello 2008). As an answer to the challenge of increasing orthopedic trauma globally, since 1999 the Surgical Implant Generation Network (SIGN) has been supplying orthopedic implants and training free of charge to over 130 hospitals in more than 50 low- and middle-income countries (Zirkle 2008). SIGN produces a solid stainless steel, interlocking intramedullary (IM) nail for the treatment of long bone factures; it can be inserted and locked without the use of an image intensifier (Ikem et al. 2007, Feibel and Zirkle 2009). Initially, re-ordering of used implants was done by mail. This was a slow and cumbersome process, and from 2003 the SIGN online surgical database (SOSD) was set up to register the surgeries done and to ease communication with SIGN surgeons worldwide (Shearer et al. 2009). To date, over 36,000 SIGN nail surgeries have been registered in the SOSD. To our knowledge, this makes the SOSD the biggest database on trauma in LMICs in the world. With the exception of some relief organizations that buy the nails from SIGN at the price of the production costs, all surgeons must report their operations to ensure re-supply of the used nails and locking screws free of charge from SIGN. There is therefore a strong incentive to register all surgeries, and the degree of reporting in 2009 was over 95% (SIGN 2011). However, reporting of follow-up carries no real incentive and Shearer et al. (2009) reported a minimum 1-month follow-up rate of only 12.6% in 2009. For this reason, some previous researchers have questioned the validity of using the SOSD for outcome measures (Shearer et al. 2009, Clough et al. 2010).

A strong argument against the use of modern orthopedic surgical trauma care, apart from the cost of the implants and the lack of personnel, has been the fear of infection. There have, however, been very few studies of good quality determining the infection rates after orthopedic surgery in low-income countries. Even though some authors have reported disturbingly high rates of postoperative infections in general and in gynecological surgery in LMICs (Reggiori et al. 1996, Eriksen et al. 2003), others have shown infection rates in orthopedic surgery matching those in high-income countries (Saris et al. 2006, Gross et al. 2010).

If it can be trusted, the huge amount of data available in the SOSD might help to give a better picture of the real risk of infection after IM nailing in LMICs. The object of this study was to describe the pattern of follow-up in the SOSD and to discuss whether the data registered—in light of the low reported follow-up rates—can be used in future in-depth research into infection rates and risk factors.

## Patients and methods

Following ethical approval by the Norwegian regional research ethics committee (20.09.10, no.2010/2040), SIGN supplied us with a data file containing an anonymous export of all surgeries registered in the SOSD from the start of the registry to October 8, 2010. The SOSD then contained surgeries involving 36,454 SIGN IM nails. 834 nails did not have the surgical approach registered. 1,228 of the nails registered involved hip, humerus, or other fracture operations. They were excluded because the numbers in each country were low, and inclusion of only tibia and femur fractures was considered more reliable for analysis. Only 2 high-income countries had registered use of SIGN nails in the SOSD. USA and Australia had registered 22 and 9 nails, respectively, and only 1 of the nails had follow-up data. Nails from these countries were therefore excluded. Remaining for analysis were 34,361 nails of the tibia or femur in 55 low- and middle-income countries with widely differing follow-up rates. Infection at follow-up in the SOSD is registered as being superficial or deep. The definition of these is at the discretion of the surgeon. Because of unclear definitions and diagnostics, and because the total infection rate was sufficient for the validation of the data in the SOSD, we did not make a distinction between the two in this study.

### Statistics

The Chi-square test was used to compare the rates of follow-up in 2 different groups. Where data were insufficient to use the Chi-square test, Fisher's exact test was used. The Student t-test was used to compare means in 2 groups. Logistic regression was used to compare rates in more than 2 groups. All p-values were 2-tailed and the level of statistical significance was set to 5% (p < 0.05). Simple descriptive statistics were used using SPSS software version 18.0.

Calculations of the follow-up rates over time were based on fixed effects in a mixed-effects Poisson regression model. The follow-up rates were analyzed using the number of follow-ups in a given time interval, and for a specific country, as a dependent variable in the analysis and the log of the total number of fractures at risk at a given time as offset in the analyses. Country was entered in the model as a random factor. Infection rates were calculated in the same way, with infection as outcome. To visualize the relation between the follow-up rates and the risk of infection, we used a generalized additive regression model (gam), with a spline smoothing of the follow-up rates compared to the risk of infection. These analyses were done using the lme4 and the mgcv libraries in the statistical program R, version 2.12.2 (R Development Core Team 2010).

## Results

The total follow-up rate (i.e. the percentage of IM nail operations with at least 1 registered follow-up visit) for all nails registered in the SOSD in October 2010 was 18.1% (CI: 17.7–18.5), and national rates ranged from 0% to 74.2%. The overall infection rate, expressed as the percentage of all registered nails that had a registered infection at follow-up, was 0.7% (CI: 0.6–0.8) for femoral fractures and 1.2% (CI: 1.0–1.4) for tibial fractures. When only nails with at least one registered follow-up visit (n = 6,224) were counted in the calculation of infection rates, the rates of infection were 3.5% (CI: 3.0–4.1) for femoral fractures and 7.3% (CI: 6.2–8.4) for tibial fractures. Countries that reported SIGN surgeries to the SOSD are listed in Table 1, along with the total number of operations registered, follow-up, and infection rates.

**Table 1.** Number of femur and tibia SIGN nail operations, follow–up, and infection rates by country in the SOSD in October 2010

Country		Nails	Follow-up			Infected	
	Bone	N	n	%	(95% CI)	%	(95% CI)
Afghanistan	Femur	893	138	16	(13–18)	1.6	(0.8–2.4)
	Tibia	698	109	16	(13–18)	1.7	(0.7–2.7)
Bangladesh	Femur	1,111	299	27	(24–30)	1.2	(0.6–1.8)
	Tibia	211	48	23	(17–28)	4.7	(1.8–7.6)
Belarus	Femur	28	1	4	(0–11)	0.0	(0.0–0.0)
	Tibia	150	5	3	(0.4–6)	0.0	(0.0–0.0)
Bhutan	Femur	39	8	21	(8–33)	2.6	(0.0–7.6)
	Tibia	126	29	23	(16–30)	1.6	(0.0–3.8)
Cambodia	Femur	2,478	550	22	(21–24)	0.7	(0.4–1.0)
	Tibia	1,587	275	17	(15–19)	0.6	(0.2–1.0)
Cameroon	Femur	309	35	11	(8–15)	0.3	(0.0–0.9)
	Tibia	116	12	10	(5–16)	1.7	(0.0–4.1)
Dominican Republic	Femur	847	22	3	(2–4)	0.5	(0.0–1.0)
	Tibia	168	4	2	(0.1–5)	0.0	(0.0–0.0)
Egypt	Femur	47	4	9	(1–17)	0.0	(0.0–0.0)
	Tibia	120	9	8	(3–12)	0.0	(0.0–0.0)
Ethiopia	Femur	347	142	41	(36–46)	1.7	(0.4–3.1)
	Tibia	139	52	37	(29–45)	2.9	(0.1–5.7)
Guatemala	Femur	320	10	3	(1–5)	0.3	(0.0–0.9)
	Tibia	200	8	4	(1–7)	1.5	(0.0–3.2)
Haiti	Femur	297	37	13	(9–16)	0.7	(0.0–1.7)
	Tibia	90	1	1	(0–3)	0.0	(0.0–0.0)
India	Femur	348	12	3	(2–5)	0.3	(0.0–0.9)
	Tibia	652	22	3	(2–5)	0.2	(0.0–0.5)
Indonesia	Femur	434	57	13	(10–16)	0.0	(0.0–0.0)
	Tibia	239	37	16	(11–20)	0.0	(0.0–0.0)
Iran	Femur	223	0	0	(0–0)	0.0	(0.0–0.0)
	Tibia	254	1	0.4	(0–1)	0.0	(0.0–0.0)
Iraq	Femur	137	69	50	(42–59)	0.7	(0.0–2.1)
	Tibia	71	38	54	(42–65)	8.5	(2.0–15)
Kenya	Femur	1,849	250	14	(12–15)	0.8	(0.4–1.2)
	Tibia	742	169	23	(20–26)	3.2	(1.9–4.5)
Malawi	Femur	236	46	20	(15–25)	1.3	(0.0–2.8)
	Tibia	66	10	15	(7–24)	1.5	(0.0–4.4)
Mongolia	Femur	229	9	4	(1–6)	0.9	(0.0–2.1)
	Tibia	306	12	4	(1–6)	0.3	(0.0–0.9)
Mozambique	Femur	131	11	8	(4–13)	0.8	(0.0–2.3)
	Tibia	12	1	8	(0–24)	0.0	(0.0–0.0)
Myanmar	Femur	1,508	343	23	(21–25)	0.7	(0.3–1.1)
	Tibia	1,234	232	19	(17–21)	1.1	(0.5–1.7)
Nepal	Femur	624	251	40	(36–44)	1.0	(0.2–1.8)
	Tibia	909	435	48	(45–51)	3.0	(1.9–4.1)
Nicaragua	Femur	165	12	7	(3–11)	0.0	(0.0–0.0)
	Tibia	238	18	7	(4–11)	0.0	(0.0–0.0)
Niger	Femur	122	16	13	(7–19)	0.0	(0.0–0.0)
	Tibia	43	6	14	(4–24)	0.0	(0.0–0.0)
Nigeria	Femur	412	49	12	(9–15)	0.0	(0.0–0.0)
	Tibia	147	23	16	(10–22)	0.0	(0.0–0.0)
Pakistan	Femur	1,493	313	21	(19–23)	0.9	(0.4–1.4)
	Tibia	1,187	203	17	(15–19)	1.2	(0.6–1.8)
Philippines	Femur	1,295	367	28	(26–31)	0.6	(0.2–1.0)
	Tibia	450	130	29	(25–33)	1.8	(0.6–3.0)
Russian Federation	Femur	380	56	15	(11–18)	0.3	(0.0–0.9)
	Tibia	420	49	12	(9–15)	0.0	(0.0–0.0)
South Africa	Femur	169	4	2	(0.1–5)	0.6	(0.0–1.8)
	Tibia	20	0	0	(0–0)	0.0	(0.0–0.0)
Swaziland	Femur	128	13	10	(5–15)	0.8	(0.0–2.3)
	Tibia	108	9	8	(3–14)	1.9	(0.0–4.5)
Tanzania	Femur	1,206	462	38	(36–41)	0.7	(0.2–1.2)
	Tibia	297	116	39	(34–45)	2.0	(0.4–3.6)
Thailand	Femur	91	25	28	(18–37)	0.0	(0.0–0.0)
	Tibia	72	8	11	(4–18)	1.4	(0.0–4.1)
Uganda	Femur	909	295	33	(30–36)	0.8	(0.2–1.4)
	Tibia	147	28	19	(13–25)	0.7	(0.0–2.1)
Vietnam	Femur	1,609	29	2	(1–3)	0.1	(0.0–0.3)
	Tibia	2,105	29	1	(1–2)	0.0	(0.0–0.0)
Countries with n < 100 **^a^**	Femur	393	99	25	(21–30)	0.8	(0.0–1.7)
	Tibia	230	62	27	(21–33)	4.3	(1.7–6.9)
Total	Femur	20,807	4,034	19.4	(18.9–19.9)	0.7	(0.6–0.8)
	Tibia	13,554	2,190	16.2	(15.6–16.8)	1.2	(1.0–1.4)
Total: femur andtibia combined		34,361	6,224	18.1	(17.7–18.5)	0.9	(0.8–1.0)

The 95% confidence intervals are based on linear calculations based on approximations to the normal distribution.

**^a^** To reduce the size of the table, all countries with less than 100 registered cases are grouped together.

No large differences in follow-up rates were seen between men and women, although we found that in Asia there was a statistically significant tendency for more women than men to return for follow-up. There were also regional differences in the proportion of female patients operated (Table 2).

**Table 2.** Total number of SIGN nails and follow-up according to sex, geographic region, and income level of country

Region / income level **^a^**	Total no.	No. of women (%)	Total no. followed up (%)	No. of females followed up (%)	p-value **^b^**
Africa	8,146	1,815 (22.3)	1,811 (22.3)	403 (22.3)	1.0
Asia	23,484	3,828 (16.3)	4,207 (17.9)	785 (18.7)	< 0.001
Latin America	2,552	390 (15.3)	200 (7.8)	26 (12.5)	0.3
Europe	179	64 (34.6)	6 (3.4)	1	0.7 **^c^**
Low-income	18,152	3,496 (19.3)	4,365 (24.1)	889 (20.4)	0.03
Lower middle-income	13,391	2,032 (15.2)	1,645 (12.3)	283 (17.2)	0.01
Higher middle-income	2,818	567 (20.1)	214 (7.6)	42 (19.6)	0.9
Total SOSD	34,361	6,095 (17.7)	6,224 (18.1)	1,214 (19.9)	< 0.001

**^a^** Income level as defined by the World Bank 2009.

**^b^** Chi-square test, gender against follow-up.

**^c^** Fisher's exact test.

Mean age at surgery in patients returning for follow-up was 33 (SD 14) years; in patients who did not have a registered follow-up it was 35 (SD 15) years (p < 0.001) (Table 3). Logistic regression analysis showed that there was a statistically significant association between increasing age and less follow-up.

**Table 3.** Follow-up for each age group compared to the < 20-year age group

Age group	n	Follow-up (%)	p-value **^a^**
< 20 years	4,237	824 (19.4)	< 0.001 **^b^**
20–29 years	11,645	2,161 (18.6)	0.2
30–39 years	7,770	1,510 (19.4)	1.0
40–49 years	4,940	902 (18.3)	0.2
50–59 years	2,823	451 (16.0)	< 0.001
≥ 60 years	2,946	376 (12.8)	< 0.001
Total	34,361	6,224 (18.1)	

**^a^** logistic regression.

**^b^** overall test.

**Figure 1.**
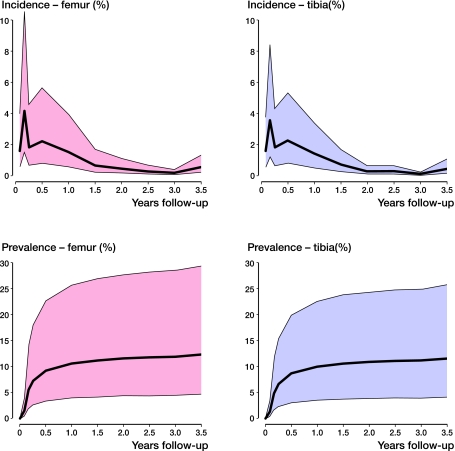
Poisson regression analysis. Pattern of follow-up rate over time for femur and tibia fractures in the SOSD. The color band signifies the 80% range of values between countries.

**Figure 2.**
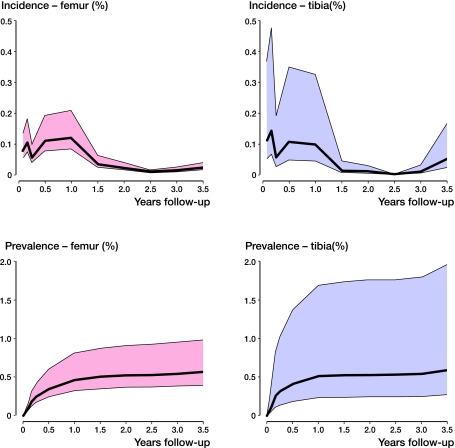
Poisson regression analysis. Pattern of infection rate for femur and tibia fractures over time in the SOSD. The color band signifies the 80% range of values between countries.

The mixed-effects Poisson regression model showed that most follow-up in the SOSD occurred in the first 2 months after surgery (Figure 1). Most infections were detected in a bimodal pattern at this time, and between 6 and 12 months after surgery (Figure 2).

**Figure 3.**
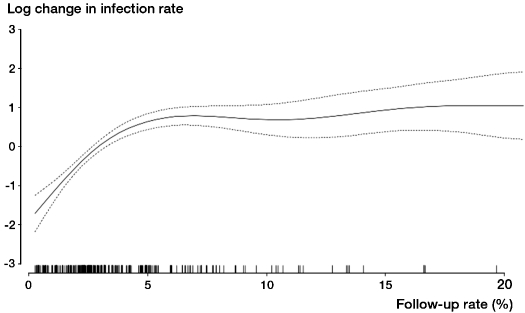
Follow-up rate plotted against log change in the infection rate. The curve is based on a generalized additive regression model (gam). Dotted lines represent 95% CI. With follow-up over 5%, there is very little increase in infection rate and the curve is consequently nearly horizontal. Short vertical lines on x-axis represent observations in different countries.

The relationship between the follow-up rates and the risk of infection, when examined in the generalized additive regression model (gam), showed that increasing national follow-up rates resulted in increasing infection rates up to a follow-up rate of approximately 5%. Follow-up rates above this did not give higher infection rates (Figure 3). This was apparent also when looking at each point in time separately.

## Discussion

Our main findings were that the infection rates in the SOSD were low and that, when we used a generalized additive regression model (gam) to look at the effects of increasing follow-up, countries with follow-up exceeding approximately 5% in the SOSD did not have statistically significantly increased infection rates with increasing follow-up. This can probably not be interpreted as if 5% follow-up in itself, in any individual center or country, is enough to catch all infections. However, it might lend support to a common notion among surgeons in low-income countries that a large proportion of people who have complaints come back for review, whereas those who do not have complaints do not return because of—among other things—the high cost of transport (Shearer et al. 2009). In some low-income countries, where large proportions of the population live on sustenance farming and have little or no cash income, many villagers will not have the money even for a local bus ticket (Gosselin 2009). It is understandable that walking many kilometers to sit in a hospital queue, sometimes for several days before being seen, may not be a high priority if people do not have a serious problem. On the other hand, a low-grade infection of an IM nail leads to pain, swelling, joint stiffness, and fistula secretion—and an acute, deep infection will make the patient very ill. In both of these situations, it is more likely that the patients will try to return to the hospital.

In a limited resource setting, one cannot expect the same follow-up rates in research as in high-income countries and a higher level of uncertainty must be accepted. If interpretation of our findings as we do above is valid, then the average national follow-up rates of approximately 18% would imply that a large (but unknown) proportion of patients with infections have returned for follow-up and in effect that the infection rates in the SOSD appear to be relatively trustworthy. However, the infection rates of 0.7% (for the femur) and 1.2% (for the tibia) in countries where the frequency of open fractures, delayed surgery, nonunions, malnutrition, and immunosuppression is known to be high may be difficult to believe for most orthopedic surgeons. When all nails without follow-up were excluded, the rates of postoperative infection were 3.5% for femoral fractures and 7.3% for tibial fractures. Even these rates are acceptable in this context, but the true infection rates probably lie somewhere between these rates. If patients with complaints really do return for follow-up more than those without complaints, this conservative estimate should be biased towards worse outcomes. On the other hand, some patients with infection are most probably lost to follow-up either because poverty forces them to live with their low-grade infection, they get treated elsewhere, or they migrate or die, and the true figures are bound to be somewhat higher than 0.7% and 1.2%. In our opinion, this is not likely to be a large proportion of patients and should not dramatically affect the estimated infection rates. There might also be situations in which patients with an infection did in fact return for follow-up, but the surgeon did not report this. Even so, the analysis did not show increased infection rates in centers where the surgeons registered more follow-up.

We believe that our findings using the above statistical model give an indication that infection rates after IM nailing in LMIC are perhaps considerably lower than many surgeons think. The overall infection rates in the SOSD are comparable to results from the literature in high-income countries (Court-Brown et al. 1992, Jenny et al. 1994, Wolinsky et al. 1999), even in the higher end of the range indicated above (Malik et al. 2004). However, most centers in high-income countries are likely to have even lower infection rates. Winquist et al. (1984) reported an infection rate of 0.9% in a series from Seattle with 520 IM nails with 17% open fractures over 25 years ago, and in a prospective series of 172 IM nail operations in Boston (Tornetta and Tiburzi 2000), no infections were seen at all.

The established perception that postoperative infection rates are high in low-income countries might be fueled by surgeons' personal experiences of the many serious infections that are encountered in an orthopedic ward in many low-income countries. However, the abundance of chronic osteomyelitis, late-presented infected open fractures, and badly done internal fixation that one can experience in these settings should not let us conclude that properly done surgery, in correctly selected patients, with modern equipment, by well trained surgeons will have poor results. The necessary basis for safe orthopedic surgery such as autoclaves, antiseptic wash, and prophylactic antibiotics has become available at most hospitals, even in the poorest countries, and hospitals that insert SIGN nails have motivated surgeons well-trained in the technique. This has been shown to be the case in general surgery in a large randomized study of prophylactic antibiotics use in Uganda, where the rate of infection after inguinal hernia repair dropped from 7.5% to 0% with correct antibiotic usage (Reggiori et al. 1996).

Even with a high prevalence of complicated cases, we really see no reason why the infection rates should not be in the same range as those in high-income countries. In fact, a prospective multicenter study comparing results of a standardized IM nailing technique between a trauma center in South Africa and Europe showed lower complication rates in South Africa and identical infection rates despite more serious injuries (Gross et al. 2010). Follow-up at 3 months in that study was 81% in South Africa and 95% in Europe. One explanation for these good results might be the lower mean age and better general health of trauma victims in South Africa. Trauma is a growing epidemic among young people in LMICs (Peden 2004, Patton et al. 2009). In the SOSD, nearly half of the patients are below the age of 30 years. The young age of the victims makes it even more important to offer modern orthopedic trauma treatment in LMICs. Perhaps it might also promise good results.

The follow-up rate in the SOSD was relatively consistent across the younger age groups, but appeared to fall off in people over 50 years. The SOSD does not contain data that can answer why this might be. One might speculate that there may be cultural reasons for this or that older people—even less than young people—are willing or have the resources to return for follow-up without having serious complaints. However, both a lower complication rate in older people with low-energy fractures and wider IM canals, and a higher mortality rate because of age related diseases, could explain this finding.

There appear to be some regional differences in follow-up patterns in the SOSD. In Africa, more than one-fifth of patients return for follow-up whereas less than 1 in 12 return for review in Latin America. In the SOSD, a marginally larger proportion of women than men return for follow-up. When stratified by regions, however, this tendency could only be seen in Asia. In Africa there was no difference in follow-up according to gender, and in Latin America there was seemingly a lower proportion of women who returned for follow-up than men. This, however, was not statistically significant with the current number of cases in the SOSD. Whether these small regional differences in women's return for follow-up are the result of cultural differences or of the economic and political state of the countries involved is not possible to answer with our study design. Stratification of countries according to income level does not appear to give more information, although the small differences seen in Table 2 were statistically significant for low-income and lower middle-income countries.

The present study had obvious limitations, the largest one being the low follow-up rate itself; which is the subject of this paper. We had to make several assumptions that may or may not be correct. We grouped superficial and deep infections together on the assumption that if they are reported, they are serious enough to be of clinical importance and we assumed that if a patient returns with a complaint it will be registered in the SOSD. All these factors introduce uncertainty into the analyses and conclusions, but we believe that the statistical models we used give strong indications that the data is complete enough to use for further studies into results and risk factors of IM nailing in LMIC. In addition, the SIGN is working hard to increase the level of follow-up. This, combined with the ever-increasing numbers in the SOSD, should help to give us more precise figures in future studies.

Very little research has been published on the results of the use of IM nails in a low-resource setting. Those studies that have been published, however, indicate that this is cost-effective treatment (Gosselin et al. 2009a) with results comparable to those found in high-income countries (Shah et al. 2004, Ikem et al. 2007, Ikpeme et al. 2011). In a world in which the growing burden of orthopedic trauma is occurring mostly in LMICs, and the safety of doing orthopedic procedures in a low-resource setting is not yet universally accepted, it is important to encourage good-quality research in order to shed light on these issues. Registry studies with large numbers of patients can demonstrate small differences in treatment outcomes sooner than smaller studies. To our knowledge, the SOSD is the largest orthopedic trauma database containing information on surgery in LMICs. It contains a wealth of information on intramedullary nail operations in over 50 countries, and presents a unique opportunity for future research to evaluate the safety and effect of orthopedic trauma surgery in general, and in low- and middle-income countries in particular. However, results from trauma registries, including the SOSD, should be confirmed by more detailed prospective studies with better follow-up. We are currently conducting such a study in Malawi.

In conclusion, it seems safe to use the data in the SOSD for studies examining infection after IM nailing in limited-resource settings, and the low infection rates in the SOSD indicate that IM nailing is a safe procedure also in low- and middle-income countries. We consider it important that more research is published on surgery in LMICs to inform policy makers and the large multilateral donors in these countries of the impact of many years of neglect of the surgical field, and the safety and good effect of modern treatment.
